# Mechanical properties of the shoulder and pectoralis major in breast cancer patients undergoing breast-conserving surgery with axillary surgery and radiotherapy

**DOI:** 10.1038/s41598-019-54100-6

**Published:** 2019-11-28

**Authors:** David B. Lipps, Joshua M. Leonardis, Robert T. Dess, Gwendolyn J. McGinnis, Robin B. Marsh, Jonathan B. Strauss, James A. Hayman, Lori J. Pierce, Reshma Jagsi

**Affiliations:** 1 School of Kinesiology, University of Michigan, Ann Arbor, MI USA; 2 Department of Biomedical Engineering, University of Michigan, Ann Arbor, MI USA; 3 Rogel Cancer Center, University of Michigan, Ann Arbor, MI USA; 4 Department of Radiation Oncology, University of Michigan, Ann Arbor, MI USA; 5 Department of Radiation Oncology, University of Texas MD Anderson Cancer Center, Houston, TX USA; 6 Department of Radiation Oncology, Northwestern University, Chicago, IL USA

**Keywords:** Outcomes research, Breast cancer, Cancer epidemiology, Biomedical engineering

## Abstract

Breast-conserving surgery (BCS) and radiotherapy reduce breast cancer recurrence but can cause functional deficits in breast cancer survivors. A cross-sectional study quantified the long-term pathophysiological impact of these treatments on biomechanical measures of shoulder stiffness and ultrasound shear wave elastography measures of the shear elastic modulus of the pectoralis major (PM). Nine node-positive patients treated with radiotherapy to the breast and regional nodes after BCS and axillary lymph node dissection (Group 1) were compared to nine node-negative patients treated with radiotherapy to the breast alone after BCS and sentinel node biopsy (Group 2) and nine healthy age-matched controls. The mean follow-up for Group 1 and Group 2 patients was 988 days and 754 days, respectively. Shoulder stiffness did not differ between the treatment groups and healthy controls (p = 0.23). The PM shear elastic modulus differed between groups (p = 0.002), with Group 1 patients exhibiting a stiffer PM than Group 2 patients (p < 0.001) and healthy controls (p = 0.027). The mean prescribed radiotherapy dose to the PM was significantly correlated with passive shear elastic modulus (p = 0.018). Breast cancer patients undergoing more extensive axillary surgery and nodal radiotherapy did not experience long-term functional deficits to shoulder integrity but did experience long-term mechanical changes of the PM.

## Introduction

Advances in modern breast cancer management have led to an increasing number of long-term breast cancer survivors in the developed world. Nearly 5 million survivors are expected to be living in the United States by 2030^1^. As a result, it is increasingly important to understand the functional consequences of treatment and to design interventions that optimize patients’ post-treatment quality of life. Impaired shoulder morbidity is a common side effect of both radiotherapy^2–4^ and axillary lymph node dissection (ALND)^5–8^. A significant subset of patients (18–46%) can experience pain, restricted shoulder mobility, stiffness, fibrosis, lymphedema and axillary web syndrome following axillary surgery and radiotherapy^2,3,9–13^.

Breast cancer patients treated with radiotherapy have a greater likelihood of poor shoulder outcomes when their radiotherapy fields are expanded to include the supraclavicular and infraclavicular nodes (regional nodes)^4^. Irradiating the regional nodes exposes a larger region of the shoulder musculature to radiation including the pectoralis major (PM)^14^. Patients treated with mastectomy and radiotherapy to the chest wall and regional nodes show compromised shoulder mobility in shoulder abduction, flexion, and internal rotation up to two years post-treatment^15^. Altered shoulder kinematics and enhanced shoulder pain continue to present six years post-treatment in patients undergoing breast cancer surgery and radiation therapy^16^. However, the expected time between treatment and the onset of measurable reductions in shoulder biomechanics vary, and may not present for 5–10 years or longer^13,17^.

Previous investigations into shoulder morbidity following radiotherapy rely on subjective patient-reported outcomes or physical assessments of strength and mobility. These methods are limited in their ability to directly assess the mechanical integrity of the shoulder and cannot isolate how specific muscles and soft tissues respond to breast cancer treatment. Robot-assisted measures of shoulder stiffness can provide insights into the overall integrity of the shoulder joint due to the stabilizing contributions of both connective soft tissues and muscles^18^. However, these techniques cannot isolate the tissue(s) that experience toxicity following treatment. Ultrasound shear wave elastography (SWE) can provide novel insights into the mechanical properties, in particular, the shear elastic modulus, of soft tissues. This technology can detect differences in muscle mechanical properties between healthy individuals and patients with neuromuscular pathologies^19–22^.

Therefore, our objective was to quantify the long-term pathophysiological impact of the combination of axillary surgery and radiotherapy on the integrity of the shoulder joint and PM of patients with breast cancer. We hypothesized that women treated with breast-conserving surgery, ALND, and radiotherapy to the breast and regional nodes would have increased shoulder stiffness and increased PM stiffness when compared to women treated with breast-conserving surgery, sentinel lymph node biopsy, and radiotherapy to the breast alone. Furthermore, we explored if observed functional deficits were correlated with the prescribed radiation dose to the PM.

## Results

### Participants

A cross-sectional design was used to detect any additional long-term shoulder morbidity exhibited in nine patients with nodal involvement who required an ALND and radiotherapy to the breast and regional nodes. These patients were compared to nine patients who had breast-conserving surgery with a sentinel node biopsy (SNB) and radiotherapy to the breast alone. Nine age-matched healthy controls with no history of breast cancer or shoulder injury were also examined.

Patient, tumor and treatment characteristics are shown in Table 1. Age, height, weight, and body mass index did not differ significantly between the two patient cohorts and healthy controls. The mean follow-up for patients who underwent breast-conserving surgery with ALND and radiotherapy to the breast and regional nodes was 988 days, which was significantly greater than the mean 754 day follow-up for patients who underwent SNB with radiotherapy to the breast alone (p = 0.003). The healthy controls were statistically stronger in vertical adduction than the two patient cohorts, but no other significant strength differences were observed between the treatment groups and controls in vertical abduction, horizontal flexion or extension, or internal or external rotation.

**Table 1:** Descriptive statistics of treatment groups and control group.

	Group 1: Breast-conserving surgery with radiation to the breast and axilla (N = 9)	Group 2: Breast-conserving surgery with radiation to the breast alone (N = 9)	Healthy controls (N = 9)	p-value
Age (years)	57 (13)	54 (11)	53 (6)	0.70
Height (cm)	165.7 (7.2)	161.2 (6.9)	166.1 (8.8)	0.34
Weight (kg)	71.7 (14.5)	66.5 (17.4)	65.5 (8.8)	0.60
BMI	26.0 (4.4)	25.7 (7.7)	23.6 (1.5)	0.57
Side Treated/Examined				
Right	5	4	8	
Left	4	5	1	
Cancer Staging				
I	0	7	—	
II	6	2	—	
III	3	0	—	
Primary Surgery				
Lumpectomy	9	9	—	
Mastectomy	0	0	—	
Axillary Surgery				
Sentinel Node Biopsy	3	9	—	
Axillary Lymph Node Dissection	9	0	—	
Number of nodes removed	17.9 (5.2)	3.8 (3.2)	—	<0.001
Chemotherapy performed?				
Yes	9	3	—	
No	0	5	—	
Days since Radiation Initiated	988 (163)	754 (111)	—	0.003
Shoulder strength (Nm)				
Vertical Adduction	46.5 (20.2)	40.9 (9.9)	61.9 (13.5)	0.019
Vertical Abduction	43.6 (21.0)	40.5 (11.7)	45.9 (12.1)	0.75
Horizontal Flexion	43.7 (13.0)	33.9 (13.4)	40.3 (12.1)	0.29
Horizontal Extension	36.1 (11.6)	26.6 (9.3)	33.6 (12.0)	0.20
Internal Rotation	24.0 (7.5)	21.7 (8.3)	30.2 (8.0)	0.088
External Rotation	25.6 (6.4)	25.9 (8.1)	31.8 (9.7)	0.23

Note: The data are reported as mean (SD) for relevant variables.

### Effect of breast cancer treatment on shoulder stiffness

**Figure 1:**
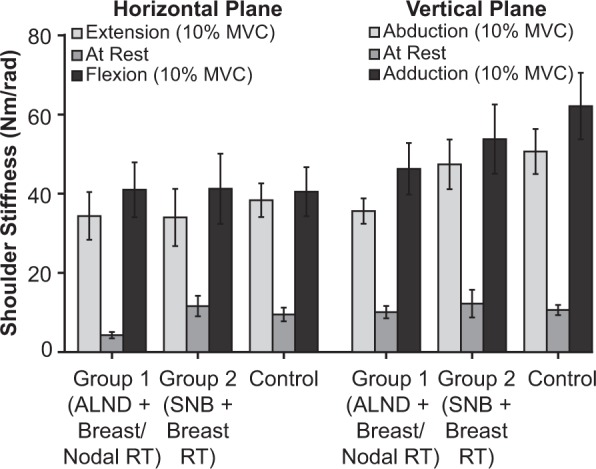
The mean shoulder stiffness in breast cancer patients previously treated with breast-conserving surgery and either sentinel lymph node biopsy and breast radiotherapy alone (n = 9) or axillary lymph node dissection and radiotherapy to the breast and regional nodes (n = 9). Participants were compared to healthy age-matched females (n = 9). Participants were examined in two movement planes while they were relaxed or generating torques scaled to 10% of their maximal strength. Error bars indicate ±1 standard error.

The overall mechanical integrity of the shoulder joint was not different between patients who underwent breast-conserving surgery with ALND and radiotherapy to the breast and regional nodes, patients who underwent breast-conserving surgery with SNB and radiotherapy to the breast alone, and healthy controls (Fig. 1). No significant treatment group differences were found for shoulder stiffness when the arm was examined in both the vertical plane (p = 0.23) and the horizontal plane (p = 0.86). There were also no significant interactions between treatment group and torque magnitude for either the vertical (p = 0.18) or horizontal (p = 0.78) planes of movement.

### Effect of breast cancer treatments on muscle elasticity

**Figure 2:**
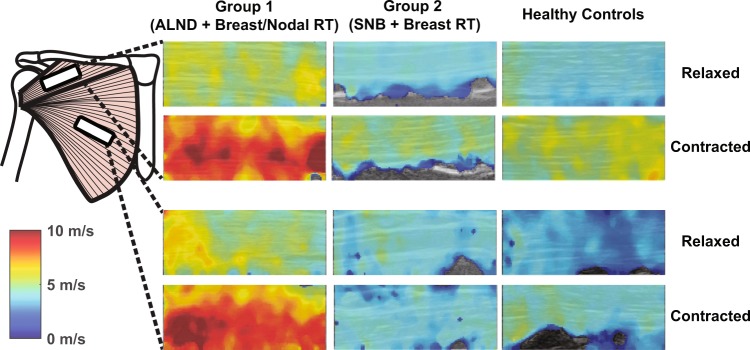
Representative ultrasound shear wave elastography maps collected from one breast + nodal RT patient (Group 1 – 1^st^ column), one breast RT only patient (Group 2 – 2^nd^ column), and one age-matched healthy control (3^rd^ column). Ultrasound images were collected from both the clavicular region (top six images) and sternocostal region (bottom six images) of the pectoralis major. In each region, the muscle was examined both in a relaxed state (top row) and contracted state (bottom row). The muscle contraction displayed here was scaled to 10% of the participant’s strength in horizontal flexion. A color scale bar is shown in the lower-left corner, indicating the measured shear wave velocity in the elastography color maps. A brighter color is indicative of a stiffer tissue.

**Figure 3:**
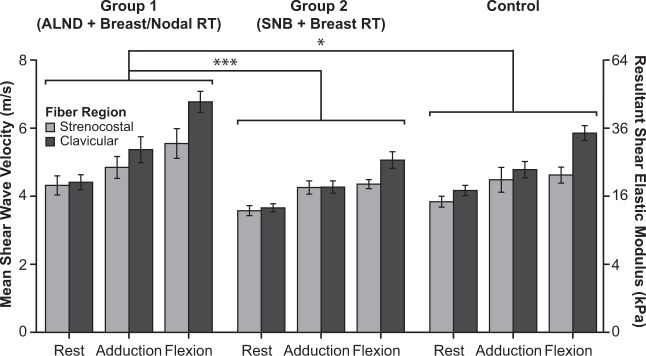
The mean shear wave velocity and resultant shear elastic modulus for the sternocostal and clavicular fiber regions of the pectoralis major in breast cancer patients previously treated with breast-conserving surgery and either sentinel lymph node biopsy and breast radiotherapy alone (n = 9) or axillary lymph node dissection and radiotherapy to the breast and regional nodes (n = 9). Both groups are compared to age-matched female controls (n = 9). The muscle was examined when patients were relaxed or generating shoulder torques in vertical adduction or horizontal flexion scaled to 10% of their maximal strength. Error bars indicate ±1 standard error.

Representative ultrasound shear wave elastography maps collected from one breast + nodal RT patient, one breast RT only patient, and one age-matched healthy control are shown in Fig. 2. Shear elastic modulus was significantly greater, indicating stiffer tissue, in patients treated with ALND and radiotherapy plans encompassing the breast and regional nodes when compared to patients treated with SNB and radiotherapy plans encompassing the breast alone (p < 0.001) and healthy controls (p = 0.027) (Fig. 3). There were no significant interactions between treatment group and muscle region (p = 0.28) or between treatment group and the three experimental conditions (p = 0.14).

### Correlation between radiotherapy dose and muscle elasticity

**Figure 4:**
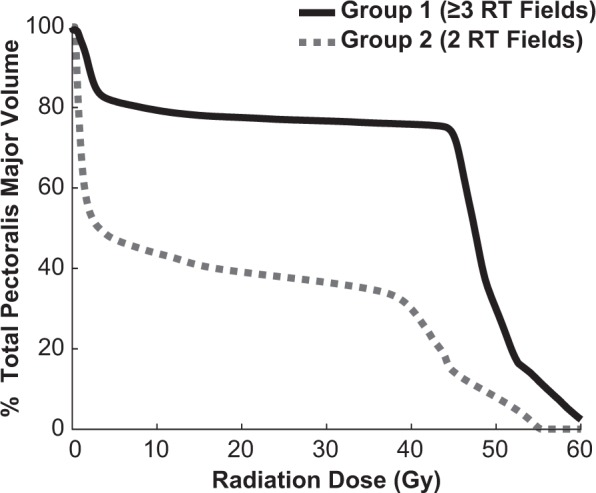
Representative cumulative dose-volume histograms for the pectoralis major muscle of a breast cancer patient treated with ≥3 RT fields (solid line) and a breast cancer patient treated with only 2 RT fields (dashed line). The value of the Y-axis represents the relative volume of the pectoralis major receiving greater than or equal to the dose on the X-axis.

Representative dose-volume histograms exhibit how radiotherapy doses differed in the PM between women treated with ≥3 RT fields and 2 RT fields (Fig. 4). The mean radiation dose delivered to the entire PM was significantly increased in women treated with ≥3 RT fields (mean (SD) 35.4 (4.2) Gy) than 2 RT fields (mean (SD) 18.9 (5.3) Gy) (p < 0.001). There was also a significant increase in the normalized volume of the PM that received at least 40 Gy (%V40 Gy) in women treated with ≥3 RT fields (mean (SD) 66.8 (8.0) %) when compared to 2 RT fields (mean (SD) 32.8 (9.7) %) (p < 0.001). The mean radiation dose to the PM was significantly correlated with the shear elastic modulus in the sternocostal region (r = 0.55, p = 0.018). Furthermore, the normalized volume of the PM receiving ≥40 Gy of radiation was significantly correlated with the shear elastic modulus of the sternocostal (r = 0.54, p = 0.021) and clavicular fiber regions (r = 0.58, p = 0.012).

## Discussion

Breast cancer survivors treated with ALND followed by radiotherapy to the breast and regional nodes did not experience additional post-treatment morbidity to the mechanical integrity of the shoulder joint when compared to patients treated with sentinel lymph node biopsy followed by radiotherapy to the breast alone. While the global integrity of the joint remained intact, we found the PM muscle was stiffer in women treated with ALND and ≥3 RT fields. This increase in muscle stiffness was positively correlated with the mean dose to the PM. These results suggest breast cancer survivors treated with breast-conserving surgery, ALND and ≥3 RT fields develop compensatory mechanisms to stabilize the joint with muscles other than the PM. The study further demonstrates the utility of ultrasound SWE for evaluating the impact of axillary surgery and radiotherapy on functional biomechanics.

The functional status of the shoulder in breast cancer survivors has relied on qualitative clinical measures of shoulder stiffness using patient-reported outcome surveys or clinical symptom grading. For example, women self-report significantly reduced shoulder stiffness when treated with hypo-fractionated nodal radiotherapy protocols over conventional fractionated radiotherapy with similar radiotherapy fields^23^. Similarly, increasing severity of shoulder stiffness using qualitative clinical grading metrics has been correlated with greater integral doses of radiation to the shoulder joint^4^. Our robot-assisted measures of shoulder stiffness provide insights into the contributions of passive soft tissues and muscle coordination to joint stability in breast cancer survivors. We found no differences in shoulder stiffness between healthy controls and patients who underwent breast-conserving surgery, axillary surgery, and radiotherapy. These findings held both as patients were examined at rest and as patients actively produced stabilizing muscle forces about the shoulder. We utilized a single posture with the arm elevated at the side, as this posture elicits the greatest contributions from the PM to shoulder function based on its muscle moment arms^24^. These quantitative measurements of shoulder stiffness highlight the overall mechanical integrity of the shoulder joint remains intact in women treated for breast cancer with RT. However, this approach alone cannot isolate if individual tissues are negatively impacted by surgery or radiotherapy.

Therefore, the current study additionally introduced the use of ultrasound SWE to obtain assessments of the mechanical response of individual muscles to breast cancer treatments. This is the first study to our knowledge to directly assess how shoulder muscles like the PM adapt to breast cancer treatments. We focused on the PM in this study as this muscle receives a moderate dose of radiation in patients treated to the breast or breast and regional nodes^14^. Only one prior study assessed functional outcomes in breast cancer survivors using ultrasound SWE^25^, and that study focused on the brachial plexus and not shoulder musculature. We were able to dissociate functional changes to the PM in women treated conservatively for breast cancer and relate these changes to the prescribed radiation dose to the muscle. The current study demonstrates the utility of this technology for assessing the PM in breast cancer survivors following surgery and radiotherapy and could support future work utilizing ultrasound SWE to screen breast cancer patients for neuromuscular deficits to the PM before they become severe, including atrophy and increased connective tissue within the muscle.

Breast cancer treatments are commonly associated with muscle disuse and atrophy^26–29^. In particular, the PM muscle atrophies following surgery and radiotherapy^29^. Additionally, we observed increases in the stiffness of the PM following breast-conserving surgery and radiotherapy. Atrophy and muscle stiffness can in part be attributed to pathogenic mechanisms in response to treatment. In particular, radiotherapy alters satellite cell production^30^, producing overproduction of collagen and extracellular matrix^31,32^. These mechanisms can limit the ability of muscle to heal and create localized regions prone to fibrosis. Functional changes to muscle, including atrophy and weakness, have been associated with radiation doses above 40 Gy^17^ and persist up to four years after radiotherapy^33^. The current study found the PM can stiffen following ALND and radiotherapy including the regional nodes, and that the increased stiffness of the PM was correlated with the prescribed radiation dose. Functionally, the observed increase in stiffness of the PM could enhance difficulties with elevating the upper extremity, which is a reported functional deficit following breast cancer treatments^15,16,34,35^. However, it is important to note that the current study did not find any significant differences in regards to the overall stiffness of the shoulder between our two patient cohorts and healthy controls, suggesting the mechanical integrity of the joint remains intact.

The current study has limitations. A small number of patients were examined, and there was some variability in treatment within each cohort. A larger study using the sophisticated measures employed in this study is necessary to confirm these findings. All participants were evaluated at least 18 months post-radiotherapy, but the pre-operative state of each patient is unknown. There were also differences in the shoulder side that was examined between the healthy controls and patient cohorts that could also influence these findings. This supports the need for a prospective study that accurately measures both the impact of axillary surgery and radiotherapy and arm dominance on shoulder and muscle stiffness relative to baseline measurements. Second, there was a difference in the days since initiating radiotherapy between the two patient groups, with the cohort that underwent radiotherapy to the breast and regional nodes being evaluated later than the cohort that underwent radiotherapy to the breast alone. Therefore, we cannot rule out an influence of the timing of assessment on some of the differences observed between these groups, although the dose-volume relationship observed is not vulnerable to timing and suggests that the differences observed did indeed relate to differences in treatment. Third, the use of chemotherapy to manage breast cancer was heterogeneous within the two patient cohorts. Chemotherapy can alter upper extremity function in breast cancer survivors^36^, but many of these symptoms are neurological^37^ and should have limited effects on our mechanical measurements. Finally, the finding of a strong correlation between radiation dose and muscle stiffness does not imply radiotherapy is the cause for these changes, particularly given differences in axillary surgery between the patient groups. Ideally, axillary surgery would be controlled by introducing an additional cohort that received axillary radiotherapy following SNB. Our institution had a relatively low volume of patients eligible for this study that received this course of treatment, and therefore this was not a feasible patient group to recruit.

In conclusion, the current study has demonstrated with robust biomechanical tools that, in our limited data set, breast cancer patients who undergo breast-conserving surgery and radiotherapy—including those who receive the most extensive surgical procedures of ALND and regional nodal radiotherapy—do not experience long-term deficits to the overall mechanical integrity of the shoulder despite reductions in vertical adduction strength. The combination of axillary surgery and radiotherapy does impact the long-term material properties of the PM, as determined with ultrasound SWE. These changes to the PM were correlated with the volume of PM treated. These findings highlight the potential use of ultrasound SWE in the future as a tool to monitor the functional status of the PM following radiotherapy for breast cancer.

## Methods

### Patients and study design

A retrospective chart review identified 40 eligible patients with breast cancer who had completed both breast-conserving surgery and radiotherapy at the University of Michigan in the previous 18–40 months. An equal number of patients who had completed radiotherapy to the breast alone or the breast and regional nodes were contacted. Letters were mailed to eligible patients. Eighteen patients agreed to participate in a single-session biomechanical assessment. All participants provided written informed consent, and all study procedures were approved by the University of Michigan Institutional Review Board (HUM00106250) and performed in accordance with the relevant guidelines and regulations. The study was approved on August 14, 2017, and participants were recruited from February 1, 2018 – May 31, 2018. Participants were excluded if they had a prior orthopedic or neurological injury of the shoulder.

A cross-sectional design was used to compare outcomes between nine node-positive patients undergoing radiotherapy to the breast and regional lymph nodes after breast-conserving surgery and ALND and nine node-negative patients undergoing radiotherapy to the breast after breast-conserving surgery and SNB. The two treatment groups were compared to nine healthy controls. The nine node-positive patients who underwent radiotherapy to the breast and regional lymph nodes were treated with three or more fields after breast-conserving surgery and ALND. For this group, regional nodal radiotherapy was delivered specifically to the supraclavicular and infraclavicular (level III) nodes in 9/9 patients, the internal mammary nodes in 6/9 patients, and the full axilla (levels I, II, and III) in 1/9 patients. A conventional fraction size of 2 Gy/day was used in all 9 patients; the dose was 50 Gy plus a boost dose to the tumor bed of 10 Gy in three patients and 46 Gy plus a tumor bed boost dose of 14 Gy in the remaining six patients. The nine node-negative patients underwent radiotherapy with two tangent beams to the breast alone (2 fields) after breast-conserving surgery and SNB. Six patients were treated with 2.66 Gy per fraction for 16 fractions to the whole breast plus a 10 Gy tumor bed boost. Two patients received 2 Gy per fraction for 25 fractions; one received a 10 Gy tumor bed boost and one did not receive a boost. The remaining patient received 2 Gy per fraction for 23 fractions plus a 14 Gy tumor bed boost. Patients received three-dimensional conformal treatment planning and radiotherapy delivery using field-in-field techniques to promote dose homogeneity.

### Robot-Assisted biomechanical measures of shoulder stiffness

**Figure 5:**
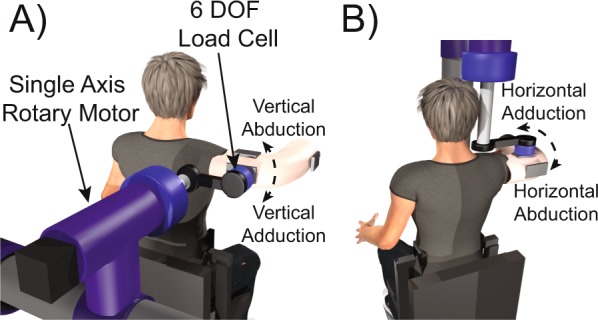
Schematic of experimental setups. A single-axis rotary motor perturbed a participant’s examined shoulder in while a six-degree-of-freedom load cell measured resultant torques in all three dimensions. Visual feedback was provided via an LCD screen. (**A**) The rotary motor was positioned to move the arm in the vertical plane while participants were relaxed or generating shoulder torques in vertical adduction (downwards) or vertical abduction (upwards). (**B**) The rotary motor was positioned to move the arm in the horizontal plane while participants were relaxed or generating shoulder torques in horizontal flexion (forward) or horizontal extension (backward). [Copyrighted figure reprinted from^38^ with permission from Springer].

The mechanical properties of the shoulder joint were quantified using robot-assisted biomechanical measures of shoulder stiffness. Participants were placed in a plastic cast that was attached to a 3-D torque sensor (JR3 Inc., Woodland, CA) and a single axis rotary motor (Baldor Electric Company, Fort Smith, AR) (Fig. 5). The arm was examined in a single posture with the shoulder elevated 90 degrees at the side. Maximum isometric shoulder strength was first measured in vertical adduction, vertical abduction, horizontal flexion, horizontal extension, internal rotation, and external rotation to scale all trials to each participant’s strength. The rotary motor was then used to evaluate the shoulder in either horizontal or vertical planes of motion. The motor rapidly moved the shoulder using a pseudorandom binary sequence with a peak amplitude of 3.4 degrees for a series of 60 seconds trials. Participants were asked to either stay relaxed or generate a torque scaled to 10% of their maximal strength in vertical ad/abduction or horizontal flexion/extension as the motor applied these perturbations. Shoulder impedance was quantified by measuring the resultant change in shoulder torque in response to a rapid change in shoulder joint angle. Impedance was then fit to a 2^nd^ order linear model in the frequency domain to describe the inertial, viscous, and stiffness properties of the shoulder^38,39^. The current study focused on stiffness, as this parameter is most indicative of a clinician’s ability to measure the resistance of the shoulder to movement during clinical examination. Six trials were performed in each measurement plane (two trials at rest and four trials generating submaximal torque), resulting in 12 total shoulder stiffness trials per participant.

### Ultrasound shear wave elastography

The material properties of the sternocostal and clavicular heads of the PM were acquired with ultrasound SWE using established protocols^21^. A Supersonic Imagine Aixplorer ultrasound machine with an SL14–5 transducer was placed on the skin above the muscle. The ultrasound technician was not blind to whether the patient had breast cancer or radiotherapy. The ultrasound machine simultaneously collects B-mode ultrasound images and a 2.5 cm × 1.0 cm elastography color map with millimeter resolution (Fig. 2). This elastography map is produced by using the same ultrasound transducer to induce acoustic shear waves in the muscle. As these shear waves propagate in a soft tissue, the transducer can record the velocity of the waves and measure the resultant shear elastic modulus using previously reported methods^40^. A faster shear wave velocity is associated with greater shear elastic modulus, and ultimately a stiffer soft tissue^41^. An established data processing algorithm in MATLAB was used to calculate the mean shear wave velocity and shear elastic modulus from the color map^21^. Images were collected from each muscle region of the PM while participants were relaxed or actively producing muscle force in horizontal flexion or vertical adduction scaled to 10% of their maximal strength. Four images were acquired for each combination of the two muscle regions and three experimental conditions, resulting in 24 total images per participant.

### Radiotherapy treatment plans

**Figure 6:**
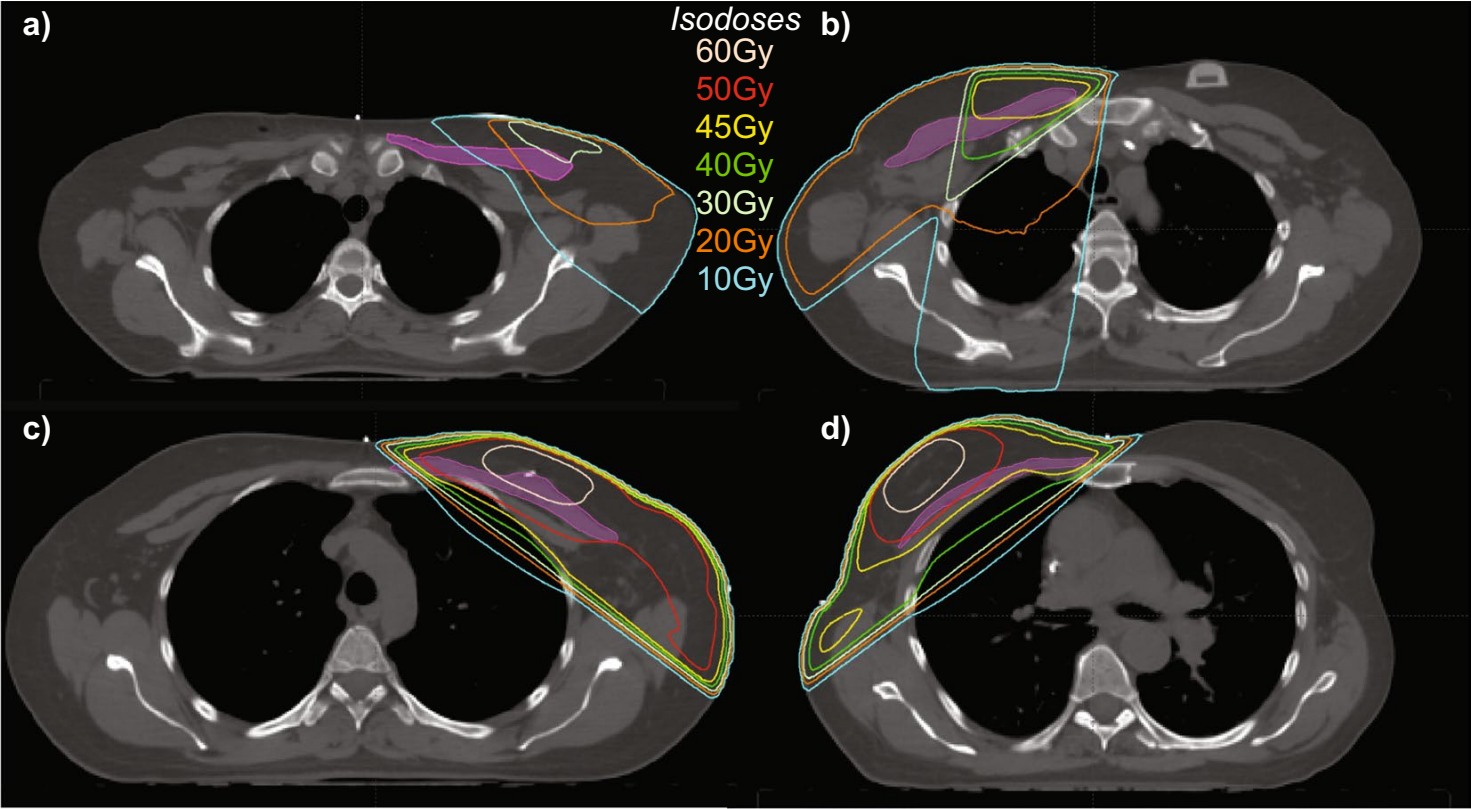
The radiation treatment plan for two representative patients are shown as isodose lines superimposed on axial computed tomography slices acquired at the T3 (**a**,**b**) and T6 (**c**,**d**) vertebrae. The treatment plans highlight differences in the amount of radiation delivered to the pectoralis major muscle (pink shaded region) differ between a breast cancer patient undergoing RT with 2 fields (**A**,**C**) and a patient undergoing RT with 3 fields (**b**,**d**).

After acquiring the radiotherapy treatment plan for each breast cancer patient using Aria (Varian Medical Systems), the PM was contoured on the original CT scans for each patient from its inferior attachment on the costal cartilage, its medial attachment on the sternum, its superior attachment on the clavicle, and its lateral attachment on the humerus (Fig. 6). The prescribed treatment plan for each patient, comprised of both the main treatment plan and the boost dose, was used to calculate the radiation dose delivered to the contoured PM muscle volume. Biologic effective dose corrections to generate equivalent 2 Gy fractions were used to account for variable fractionation schemes (assuming alpha/beta ratio of 2.5^42^). Both the mean dose (in Gy) and the normalized PM volume receiving greater than 40 Gy (%V40Gy) were computed.

### Statistical analysis

Statistical analyses were performed in SAS 9.4 (SAS Institute Inc, Cary, NC). Differences in patient demographics, treatment plans, and maximal isometric strength between the two treatment groups and healthy controls were tested using the PROC ANOVA procedure. Our first hypothesis that women treated with breast-conserving surgery, ALND, and radiotherapy to the breast and regional nodes would have increased shoulder stiffness was examined using a linear mixed effects model (PROC MIXED procedure). Shoulder stiffness was treated as the outcome measure, treatment group (three levels: two breast cancer cohorts and healthy controls) was treated as a between-subjects factor, movement plane (two levels: vertical or horizontal plane) and torque magnitude (three levels: relaxed, 10%, and −10% MVC) were treated as within-subjects factors, and subjects were treated as a random factor. Our second hypothesis that women treated with breast-conserving surgery, ALND, and radiotherapy to the breast and regional nodes would have increased PM stiffness was tested using a similar linear mixed effects model framework. Mean shear wave velocity (which is associated with tissue elasticity) was our primary outcome measure, treatment group was treated as a between-subjects factor, experimental condition (three levels: relaxed or generating 10% MVC in horizontal flexion, or vertical adduction) and PM fiber region (two levels: sternocostal or clavicular region) were treated as within-subjects factors, and subjects were a random factor. Significant main effects and interactions were further examined with Tukey-corrected posthoc comparisons. For the treatment groups only, independent t-tests were performed to identify differences between the radiation treatment plans between the two cohorts. The primary outcome variables for these t-tests were the mean radiation dose or %V40Gy. Pearson correlations compared the shear wave velocity of each fiber region of the PM at rest to the mean radiation dose or %V40Gy.

## Data Availability

The datasets generated and analyzed during the current study are available in a public repository at 10.6084/m9.figshare.8870798.
